# Structural Evolution of Two-Phase Blends of Polycarbonate and PMMA by Simultaneous Biaxial Stretching

**DOI:** 10.3390/polym10090950

**Published:** 2018-08-27

**Authors:** Takumi Kobayashi, Hiromu Saito

**Affiliations:** Department of Organic and Polymer Materials Chemistry, Tokyo University of Agriculture and Technology, Koganei-shi, Tokyo 184-8588, Japan; takumi-kobayashi@pentel.co.jp

**Keywords:** biaxial stretching, blend, polycarbonate, PMMA, phase inversion, surface hardness

## Abstract

We investigated the structural evolution of the two-phase blends of polycarbonate (PC) and poly(methyl methacrylate) (PMMA) at various blend compositions by simultaneous biaxial stretching, using optical microscopy and SEM observation. The spherical PMMA domains and PC matrix of 30/70 PC/PMMA were enlarged uniformly at the all in-plane direction, while the anisotropic-shaped co-continuous structure in 50/50 PC/PMMA was deformed to a crosshatched structure by the in-plane bimodal orientation. In 70/30 PC/PMMA, the phase inversion was found to occur by simultaneous biaxial stretching; that is, the spherical PMMA domains were changed to a crosshatched matrix by the in-plane bimodal orientation due to coalescence of the PMMA domains during the stretching. Owing to the phase inversion, the surface hardness estimated by the pencil hardness test became harder, from 2B to 2H, increasing the strain from 1.0 to 2.0.

## 1. Introduction

Biaxially stretched polymer films are often used as packaging materials for food and industrial products, because the mechanical and gas barrier properties can be improved using biaxial stretching in the manufacturing process [1]. Many researchers have investigated how the structures of polymer films obtained by biaxial stretching vary in a number of aspects [2,3] for crystalline polymers [4,5,6,7,8,9,10,11,12,13,14,15,16,17,18,19,20,21,22,23,24,25,26,27], polymer blends [28,29,30,31,32,33,34] and composites [35,36,37]. The dimensional stability is enhanced due to the increase in the degree of the stress-induced crystallinity [4], surface morphology is changed [5,6], hardness is enhanced [7], and high toughness polylactide film can be obtained by development of highly-oriented small crystallites [8] in the crystalline polymers. The mechanical property is improved in the uncompatibilized polymer blends by addition of the interfacial modifier due to suppression of the interfacial voiding [28], oxygen barrier property is improved in the multi-layered polypropylene (PP)/polyethylene oxide system [29], and microporous structure is developed in the PP/nylon 6 blends [30]. The silica nanofillers are elongated parallel or perpendicular to the deformation directions depending on the type of nanofiller [35] and biaxial stretching of a PP/clay nanocomposite results in delamination and orientation of clay stacks [36,37]. Orientation by biaxial stretching is characterized by wide-angle x-ray diffraction [4,9,10,11,12,13,14,15,16,17,28,31,32], birefringence [9,13,14,15,16,17,18,19,20], infrared spectroscopy [13,20,21,22,23], fluorescence anisotropy [24], stress-strain behavior [14,16,17,25,26,27,32,33]. Isotropic plane film can be produced by simultaneous biaxial stretching, in which films are stretched in the X and Y directions at the same time, so that the properties of the stretched specimen thus produced are isotropic in the in-plane direction [14,16,32]. This indicates that a uniform orientation at all in-plane direction is caused by simultaneous biaxial stretching; that is, the polymer chains and crystalline lamellae are uniformly oriented at all in-plane direction. The in-situ polarized infrared spectroscopic study by Nitta et al. suggests that the molecular chain is elongated uniformly at the all in-plane direction by simultaneous biaxial stretching of PP [20,23]. Though the uniform orientation without the preferred orientation is considered in the simultaneous biaxial stretching, the bimodal orientation to the stretching directions of X and Y is also suggested by a wide angle x-ray diffraction study in crystalline polymers such as polyethylene terephthalate (PET) and polyethylene naphthalate [16,31]. However, it is difficult to clarify the bimodal orientation of the structure in neat polymers because of the difficulty in observing the stretched structure.

Bisphenol-A polycarbonate (PC) is a ductile material. Because of its excellent mechanical properties, PC is used for various goods such as window glass, bottles, bulletproof glass, and more. Although it has high impact-resistance, it exhibits low scratch-resistance due to low surface hardness; its pencil hardness is 4B. To improve the scratch-resistance, hard coatings are applied on the PC surface by physical or chemical vapor deposition [38] and sol-gel methods [39,40]. On the other hand, poly(methyl methacrylate) (PMMA) has poor toughness, but exhibits high scratch-resistance due to high surface hardness; its pencil hardness is 4H. The tensile property of glassy PMMA changes from brittle to ductile with increasing temperature [41], while that of glassy PC changes from ductile to brittle when the molecular weight is low [42,43]. Fracture toughness of PMMA and PC abruptly increases at very high loading rates [44]. Yamaguchi et al. found that the surface hardness of PC could be enhanced by blending PMMA, due to the surface localization of PMMA by segregation during the injection-molding. The durometer D hardness increased from 80 to 83 by blending 5% of PMMA [45]. The segregation of PMMA might be attributed to the orientation-induced phase separation resulting from variations in viscosity, due to composition fluctuations [46,47]. A PC/PMMA blend is partially miscible and is suggested to exhibit a lower critical solution temperature (LCST) type phase diagram, in which liquid–liquid phase separation occurs at a temperature above the LCST [48,49]. When a blend has a two-phase structure, the spherical domain dispersed in the matrix deforms to an ellipsoidal one by uniaxial elongational flow [50,51,52], and the deformed structure recovers to a spherical one by relaxation after the flow stops [52]. A fibrillar shaped two-phase structure is also obtained by the extrusion process [53,54,55,56,57]. Owing to the structural color induced by the fibrillar shaped two-phase structure, iridescent luster is generated by the uniaxial elongation of 70/30 PC/PMMA.

Our interest in this study is the structural evolution of two-phase blends of PC and PMMA by simultaneous biaxial stretching, and the change of the surface hardness achieved by this stretching. To observe the stretched structure obtained by simultaneous biaxial stretching, we investigated the evolution of the two-phase structure of the PC/PMMA blends by using optical microscopy and SEM observation. When the two-phase structure obtained after simultaneous stretching is isotropic, the domain structure is enlarged uniformly at the all in-plane direction, for instance. We chose blends of PC and PMMA because various two-phase structures with a size in a micrometer scale can be obtained at different blend compositions. For example, the spherical domain structure is obtained at a 30/70 composition, while a co-continuous structure is obtained at a 50/50 composition. Hence, various structural evolutions by simultaneous biaxial stretching can be observed by microscopic observation. The result of the surface hardness estimated by the pencil hardness test is also presented, in order to discuss the structure change achieved by stretching. The control of the surface hardness is significant for the application of the packaging materials.

## 2. Materials and Methods

The PC and PMMA specimens used in this study were commercial polymers. The PC was bisphenol-A polycarbonate supplied by the Mitsubishi Gas Chemical Company, Inc. (Tokyo, Japan); Iupilon S-2000 N, *M*_w_ = 2.4 × 10^4^ g mol^−1^. The PMMA was supplied by Kurarey Co., Ltd. (Tokyo, Japan); Parapet G, *M*_w_ = 1.0 × 10^5^ g mol^−1^. The glass transition temperatures (*T*_g_s) of PC and PMMA were 150 °C and 100 °C, respectively.

The PC and PMMA were melt-mixed at a temperature of 250 °C and at a rotor speed of 90 rpm for 5 min in a mixing chamber of a miniature mixing machine (Imoto IMC-18D7, Kyoto, Japan). The melt mixed blend was extruded and chopped into pellets. The pellets were then melt pressed in a vacuum hot-press machine (Imoto IMC-11FD, Kyoto, Japan) at 250 °C and 10 MPa for 5 min, to obtain the unstretched film with a thickness of about 100 µm, which was then cooled to room temperature. The PC and the blends used in this study did not show crystallization-and-melting behavior in these experimental conditions. To erase the effect of local ordering of PC [58], the PC blends were melt-pressed at 250 °C, which was too high compared with the *T*_g_ of PC and PMMA. The unstretched film was cut into square-shaped specimens of 65 mm × 65 mm to undergo the biaxial stretching.

**Figure 1:**
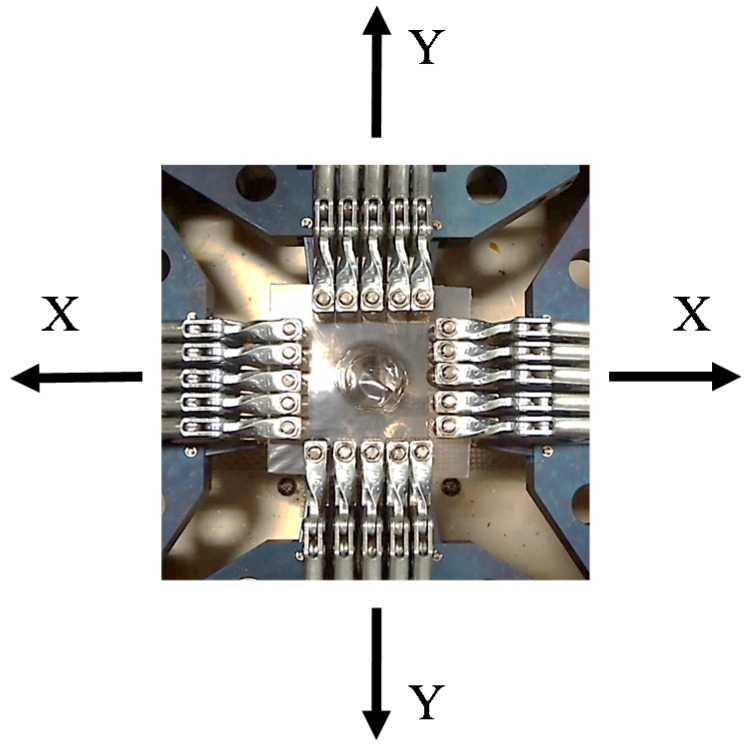
Photograph of clamps in a biaxial stretcher.

The biaxial stretching was performed by a biaxial stretcher equipped with a temperature controller (IMC-1A94, Imoto Machinery Co., Ltd., Kyoto, Japan). The film specimen was gripped on each side by two pairs of five clamps (Figure 1), and the initial gauge area before stretching was 50 mm × 50 mm. The specimen was heated and then biaxially stretched at 140 °C for 30/70 and 50/50 PC/PMMA, 160 °C for 70/30 PC/PMMA, and 180 °C for neat PC after maintaining the temperature for 5 min. These temperatures were the most suitable for stretching uniformly at the all in-plane direction. The biaxial stretching was carried out at a draw speed of 100 mm min^−1^ with various draw ratios, by moving the clamps simultaneously in two stretching directions (the X and Y directions, as shown by arrows in Figure 1) at the same speed and to the same draw ratio. Then, the stretched specimen was cooled to room temperature for the observation.

The phase structure of the blends was observed both under an unpolarized optical microscope and a polarized optical microscope (Olympus BX53-P, Tokyo, Japan), each equipped with a charge-coupled device (CCD) camera (Olympus DP73, Tokyo, Japan). The structure under the polarized optical microscope was observed by the optical microscope equipped with a sensitive tint plate, with an optical path difference of 530 nm under crossed polarizers P and A. Here, the optical axis of the polarizer was rotated at 45° to the stretching directions of X and Y, and that of the sensitive tint plate (ST).

The phase structure of the blend was also observed under a scanning electron microscope (SEM) (Hitachi S2100A, Tokyo, Japan). In order to observe the surface and cross section of the blend specimen, the PMMA component was etched by 2-butanone for 3 h at 20 °C, and the PC component was etched by sodium hydroxide (NaOH) solution (30 wt % NaOH) for 48 h at 20 °C. The etched specimen was sputter coated with platinum using a sputter system (JEOL JFC-1300, Tokyo, Japan). After the conductive coating, SEM was operated with an accelerating voltage of 20 kV at 20 °C under vacuum.

The surface hardness of the blend specimen was estimated by the pencil hardness test according to ISO 15184. A graphite pencil was used to draw on the film specimen with uniform pressure, maintaining the pencil at a constant angle of 45°. The surface hardness was determined using pencils of various degrees of hardness by judging whether an indentation was caused or not; the specimen was judged as harder than the pencil hardness when no indentation was seen.

## 3. Results and Discussion

**Figure 2:**
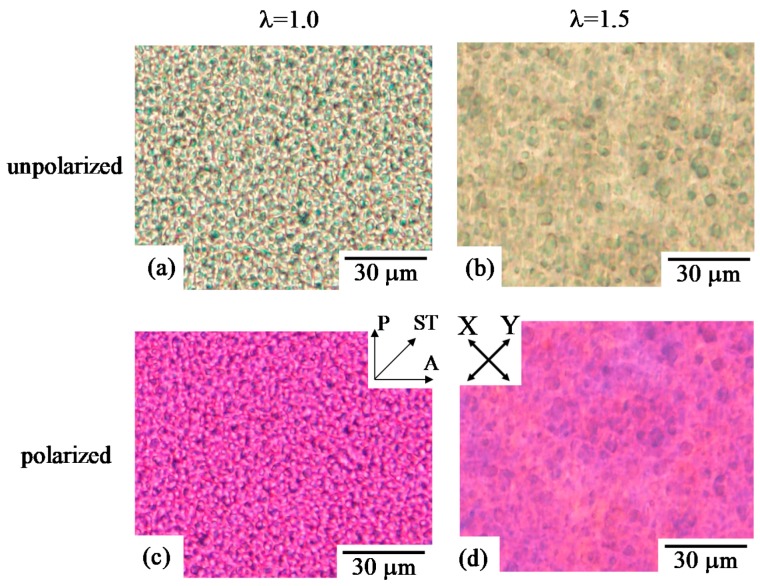
Unpolarized and polarized optical micrographs of 30/70 polycarbonate/poly(methyl methacrylate) (PC/PMMA) obtained by simultaneous biaxial stretching at draw ratios λ of 1.0 and 1.5. (**a**) λ = 1.0 (unpolarized); (**b**) λ = 1.5 (unpolarized); (**c**) λ = 1.0 (polarized); (**d**) λ = 1.5 (polarized).

Figure 2 shows unpolarized and polarized optical micrographs of 30/70 polycarbonate (PC)/poly(methyl methacrylate) (PMMA) obtained by simultaneous biaxial stretching at a draw ratio λ of 1.5 (λ = 1.5), and the unstretched blend (λ = 1.0). Here, the specimen was stretched at 140 °C. The blend specimen in this composition was fractured at a draw ratio above 1.5. Spherical PC domains with diameters of several micrometers were dispersed in the PMMA matrix at λ = 1.0 (Figure 2a). The spherical domain became larger without a change of the shape, and the domain distance became longer by simultaneous biaxial stretching (Figure 2b), indicating that the two-phase structure of the spherical domain and matrix is enlarged uniformly at the all in-plane direction. PC can be elongated to a high strain [59], though the stretching temperature of 140 °C was below the *T*_g_ of the PC (150 °C); meaning that the crazing structure and debonding at the interphase were not observed at λ = 1.5. In spite of the enlargement of the two-phase structure, the change of the interference color due to stretching was small and the structure was unclear under polarized optical microscopy (Figure 2d), indicating that the deformed structure was optically isotropic. If the deformation is truly biaxial, it is expected to be optically isotropic in the plane of the film. Thus, the results suggest that the two-phase structure of the spherical domain and matrix is elongated uniformly at the all in-plane direction, without the preferred orientation, by simultaneous biaxial stretching. These results are consistent with those demonstrated in neat polymers such as polypropylene, poly(lactic acid) and poly(ethylene terephthalate), in which the stretched specimen is optically isotropic due to uniform deformation without the preferred in-plane orientation by simultaneous biaxial stretching [14,16,32].

**Figure 3:**
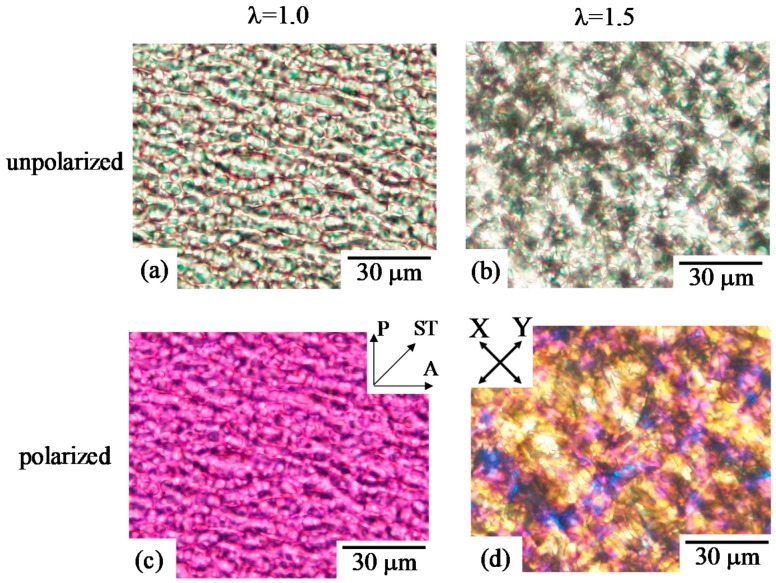
Unpolarized and polarized optical micrographs of 50/50 PC/PMMA obtained by simultaneous biaxial stretching at draw ratios λ of 1.0 and 1.5. (**a**) λ = 1.0 (unpolarized); (**b**) λ = 1.5 (unpolarized); (**c**) λ = 1.0 (polarized); (**d**) λ = 1.5 (polarized).

A co-continuous two-phase structure was obtained in 50/50 PC/PMMA. The change of the co-continuous two-phase structure by simultaneous biaxial stretching is shown in Figure 3. The blend specimen in this composition was fractured at a draw ratio above 1.5. The size of the co-continuous structure became larger by stretching (Figure 3b). The crazing structure and debonding at the interphase were not observed at λ = 1.5. The interesting result here is that blue and yellow interference colors appeared, and a crosshatched pattern was seen in the stretched blend under the polarized optical microscopy (Figure 3d), indicating that the component phase is optically anisotropic and the anisotropic-shaped phase is formed along the crosshatched structure. The change and enlargement of the phase structure might be attributed to the evolution of the liquid–liquid phase separation, as discussed later in the results of 70/30 PC/PMMA. Blue and yellow interference colors were seen along the crosshatched structure, which was elongated to the X and Y stretching directions. These results suggest that the anisotropic-shaped domain is deformed to the X and Y stretching directions by an in-plane bimodal orientation to yield the crosshatched structure.

**Figure 4:**
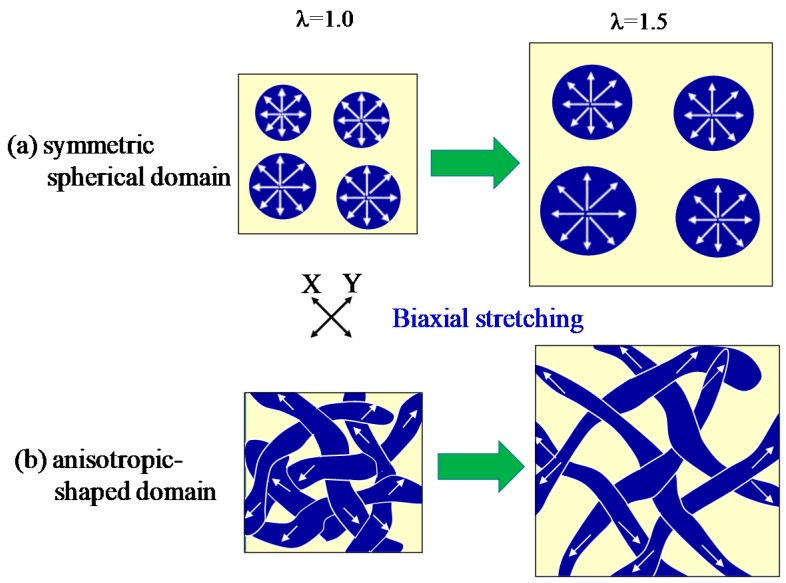
Schematic illustration for the deformation direction of the two-phase structure by simultaneous biaxial stretching. (**a**) symmetric spherical domain; (**b**) anisotropic-shaped domain.

Figure 4 shows the schematic illustration for the deformation of the two-phase structure of PC/PMMA blends by simultaneous biaxial stretching. The symmetric spherical domain is deformed uniformly at the all in-plane direction, without the preferred orientation by stretching (Figure 4a). On the other hand, the co-continuous two-phase structure consists of an unsymmetrical anisotropic-shaped domain, and the long axis of the anisotropic shaped domain is rotated to the X and Y stretching directions, with the domains deformed by the in-plane bimodal orientation (Figure 4b). 

**Figure 5:**
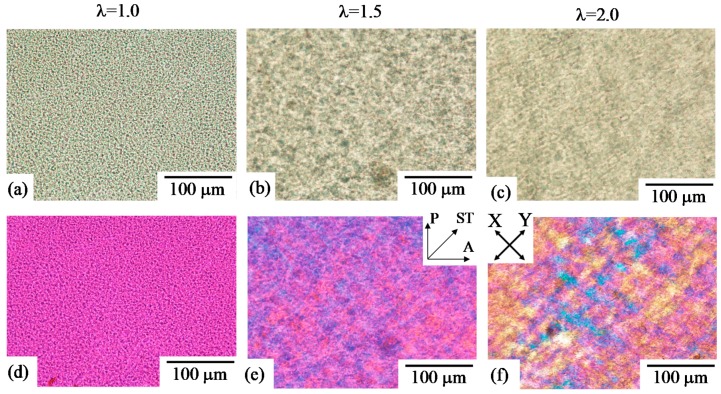
Unpolarized and polarized optical micrographs of 70/30 PC/PMMA obtained by simultaneous biaxial stretching at various draw ratios λ. The upper pictures are optical micrographs, and the lower pictures are polarized optical micrographs. (**a**) λ = 1.0 (unpolarized); (**b**) λ = 1.5 (unpolarized); (**c**) λ = 2.0 (unpolarized); (**d**) λ = 1.0 (polarized); (**e**) λ = 1.5 (polarized); (**f**) λ = 2.0 (polarized).

In 70/30 PC/PMMA, the characteristic change of the two-phase structure occurred as shown in Figure 5. Here, the specimen was stretched at 160 °C. The two-phase structure of the PMMA spherical domains dispersed in the PC matrix was seen in the unstretched blend (Figure 5a). Though the phase structure with spherical domains was similar to that observed in Figure 2 for 30/70 PC/PMMA, the structural evolution by stretching was quite different in 70/30 PC/PMMA. The spherical shape of the PMMA domains changed to the anisotropic-shaped domain (Figure 5b,c). Blue and yellow interference colors appeared, and a crosshatched pattern was seen along the X and Y stretching directions under polarized optical microscopy (Figure 5e,f). The results indicate that the component phase is optically anisotropic, and the anisotropic-shaped phase is formed along the crosshatched structure by the in-plane bimodal orientation, though the deformed structure was optically isotropic and no change of the spherical shape was seen in 30/70 PC/PMMA by stretching at 140 °C, which was below the *T*_g_ of PC (150 °C).

**Figure 6:**
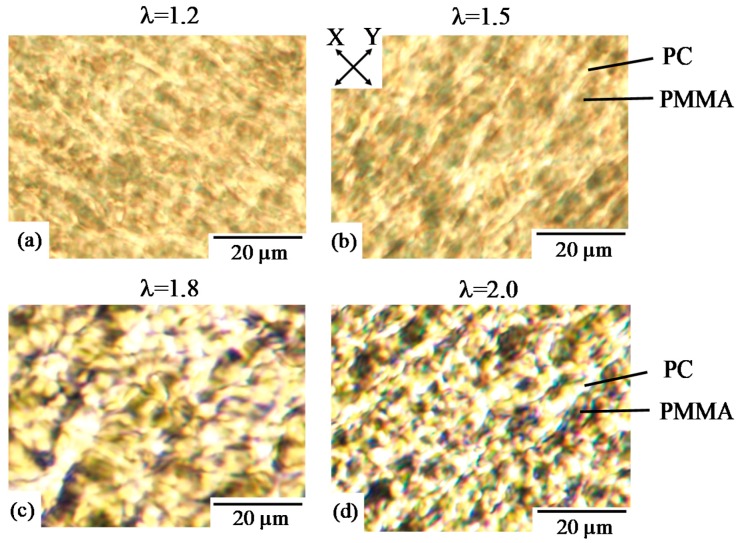
Unpolarized optical micrographs of 70/30 PC/PMMA obtained by simultaneous biaxial stretching at various draw ratios λ. (**a**) λ = 1.2; (**b**) λ = 1.5; (**c**) λ = 1.8; (**d**) λ = 2.0.

To understand the evolution of the phase structure during the simultaneous biaxial stretching of 70/30 PC/PMMA, the magnified images for the unpolarized optical microscopies are shown in Figure 6. The PMMA spherical domains shown in Figure 5a were coalesced and aggregated to yield large anisotropic-shaped distorted domains, and the PC matrix was deformed to a fibrillar structure in the X and Y stretching directions by the in-plane bimodal orientation (Figure 6a,b). The deformation of the PC matrix by the in-plane bimodal orientation might be caused by the rotation of the anisotropic-shaped PMMA domains to the X and Y stretching directions. The phase structure became larger with increasing λ, and a continuous PMMA phase was seen (Figure 6c,d). The change and enlargement of the phase structure observed in 70/30 PC/PMMA might be attributed to the evolution of the liquid–liquid phase separation by stretching at 160 °C, which was above the *T*_g_s of both component polymers. Since PC/PMMA blends are partially miscible and are suggested to exhibit an LCST type phase diagram [48,49], the evolution of the liquid–liquid phase of separation might be caused by shifting the LCST phase diagram to a lower temperature by stretching [60] and the orientation-induced phase separation [46,47,61].

**Figure 7:**
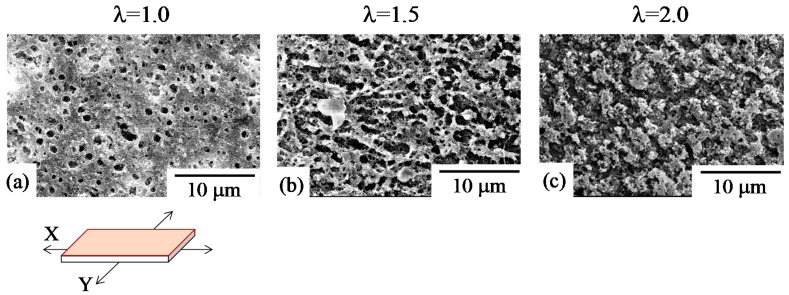
SEM micrographs of the surface of 70/30 PC/PMMA obtained by simultaneous biaxial stretching at various draw ratios λ. (**a**) λ = 1.0; (**b**) λ = 1.5; (**c**) λ = 2.0.

Figure 7 shows SEM micrographs of the surface of 70/30 PC/PMMA obtained by simultaneous biaxial stretching, which correspond to the optical micrographs shown in Figure 5 and Figure 6. Since SEM observation was carried out after extraction of the PMMA phase by etching with 2-butanone, the remaining material was PC. Thus, spherical holes with a diameter of several micrometers (seen in Figure 7a) are assigned to the PMMA domains, while the white matrix is assigned to the PC matrix. This indicates that the PMMA domain is dispersed in the PC matrix in the unstretched blend. The spherical domain of PMMA changed to ellipsoidal domain, and the aspect ratio increased in the X and Y direction with an increasing draw ratio (Figure 7b). This result supports the result demonstrated in Figure 5 and Figure 6; that spherical PMMA domains are elongated to the X and Y stretching directions by the bimodal in-plane orientation. At λ = 2.0, a crosshatched network structure was formed in the PMMA phase (Figure 7c). That is, phase inversion occurred by simultaneous biaxial stretching—the PMMA spherical domain was inverted to the crosshatched network matrix. To our knowledge, this is the first study to observe the phase inversion of two-phase polymer blends by simultaneous biaxial stretching. The phase inversion might be caused by the coalescence and aggregation of PMMA domains owing to Ostwald ripening [62], due to the evolution of the liquid–liquid phase separation by stretching, as demonstrated in the following SEM micrographs.

**Figure 8:**
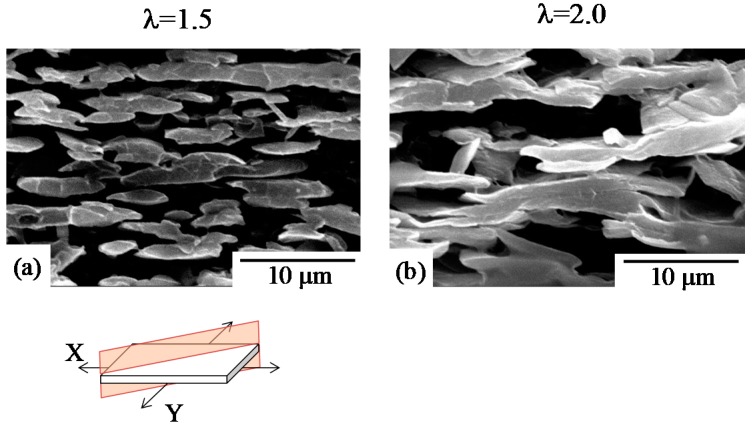
SEM micrographs of the cross section of 70/30 PC/PMMA obtained by simultaneous biaxial stretching at draw ratios λ of 1.5 and 2.0. (**a**) λ = 1.5; (**b**) λ = 2.0.

Figure 8 shows SEM micrographs of the cross section of 70/30 PC/PMMA. Since the SEM observation was carried out after extraction of the PC phase by etching with NaOH, the remaining material was PMMA. The spherical domain of PMMA changed to an ellipsoidal or fibrillar domain, in which the major axis of the domain is parallel to the plane of the films by stretching (Figure 8a). This is consistent with the in-plane orientation and stretch-thinning in the normal thickness direction suggested in the simultaneously biaxial stretched films [14,16,32]. The PMMA domains were then aggregated in the thickness direction, and the elongated domains became thicker in the thickness direction by the biaxial stretching (Figure 8b). These results suggest that coalescence and aggregation of PMMA domains occurred by simultaneous biaxial stretching. Thus, phase inversion from the PMMA domain to the PMMA matrix by simultaneous biaxial stretching, demonstrated in Figure 6, is attributed to the coalescence and aggregation of the PMMA domains. Such coalescence and aggregation of the PMMA domains might be caused by Ostwald ripening [62], due to the evolution of the liquid–liquid phase separation by stretching.

**Figure 9:**
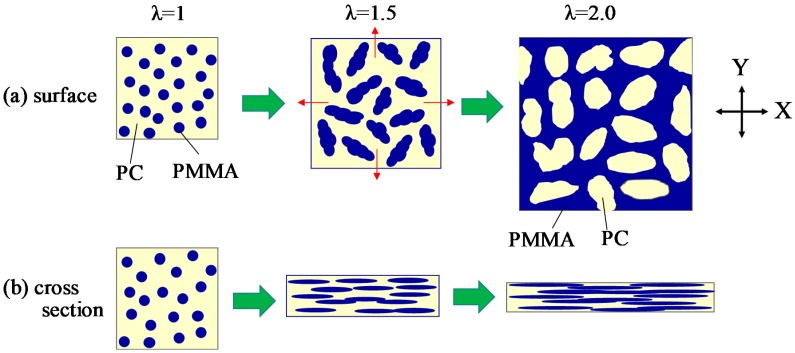
Schematic illustration for the structure change of 70/30 PC/PMMA by simultaneous biaxial stretching. (**a**) surface; (**b**) cross section.

Figure 9 shows the schematic illustration for the structural evolution of 70/30 PC/PMMA during simultaneously biaxial stretching. The spherical PMMA domains dispersed in the PC matrix are coalesced and aggregated by Ostwald ripening due to the evolution of the liquid–liquid phase separation by stretching, and the anisotropic-shaped aggregated domains are formed. The long axis of the anisotropic-shaped PMMA domain is rotated to the X and Y stretching directions by the in-plane bimodal orientation, as suggested in Figure 4b (λ = 1.5: Figure 9a). Owing to the existence of the anisotropic-shaped PMMA domain, the PC matrix is deformed by the in-plane bimodal orientation. Due to subsequent coalescence and deformation of the anisotropic-shaped PMMA domains, the PMMA domains are inverted to a crosshatched matrix (λ = 2.0: Figure 9a). During the coalescence and deformation in the X and Y stretching directions, the anisotropic-shaped PMMA domain is rotated in the in-plane projection and is squashed in the thickness direction by stretch-thinning (Figure 9b).

**Figure 10:**
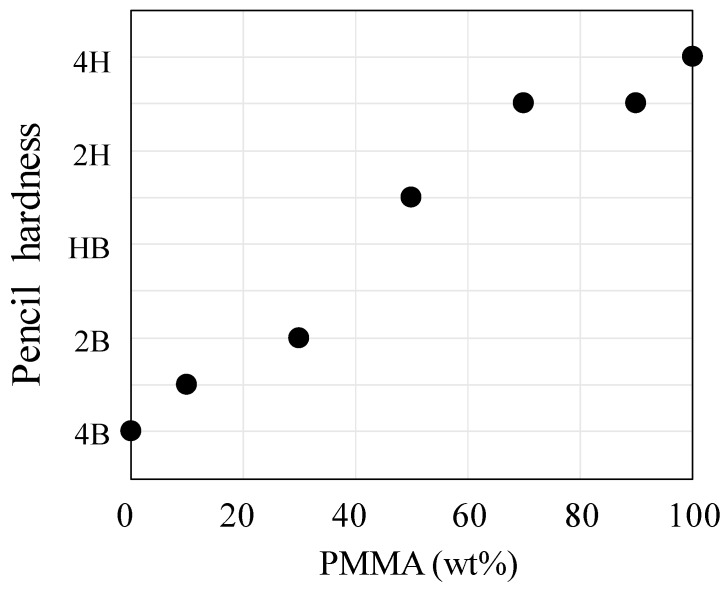
Surface hardness of unstretched PC/PMMA blends at various compositions.

Figure 10 shows the surface hardness of the unstretched PC/PMMA films at various blend compositions. Here, the surface hardness was estimated by the pencil hardness test. The surface hardness of neat PC and neat PMMA was 4B and 4H, respectively. The surface hardness becomes harder, from 4B to 4H with increasing the PMMA composition. As shown in Figure 2 and Figure 5, the matrix in 30/70 PC/PMMA and 70/30 PC/PMMA was PMMA and PC, respectively. Hence, the surface hardness of the blends became harder by increasing the amount of PMMA at the surface, with the increase of the composition of PMMA. The change of the pencil hardness was large at around a PMMA composition of 50 wt %, in which a co-continuous structure was formed, suggesting that the critical composition for the change of the phase is estimated to be 50 wt %. On the other hand, the change of the pencil hardness was small at the PMMA composition above 70%, suggesting the existence of large amount of PMMA at the surface due to the surface localization at the PMMA composition above 70%.

**Figure 11:**
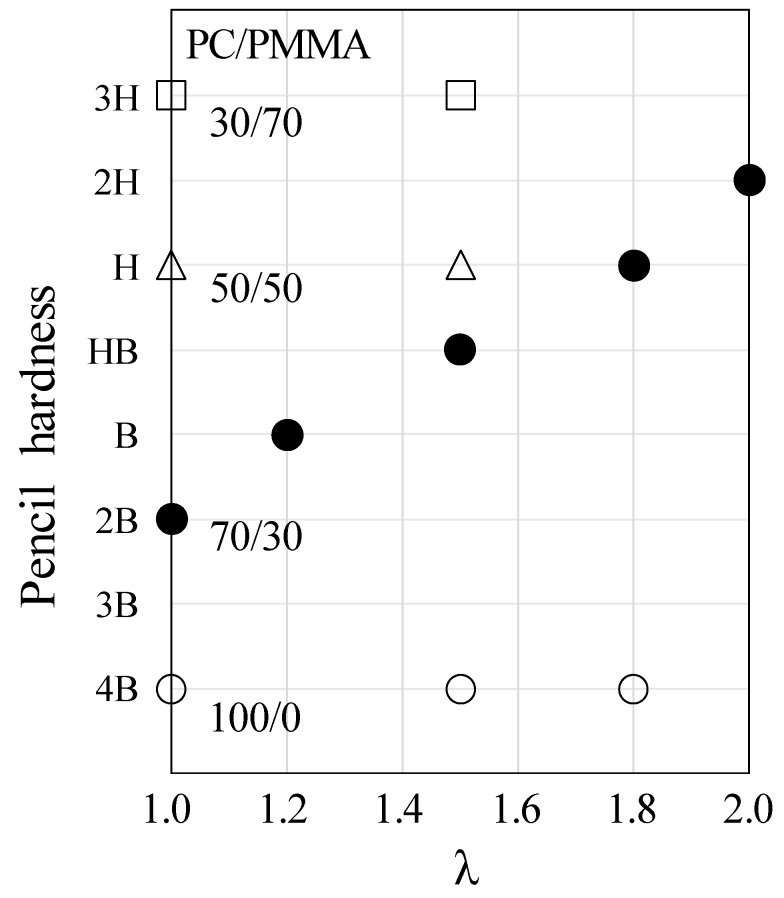
Surface hardness of PC/PMMA blends obtained by simultaneous biaxial stretching at various draw ratios λ. ◯: 100/0 PC/PMMA; ●: 70/30 PC/PMMA; △: 50/50 PC/PMMA; □: 30/70 PC/PMMA.

Figure 11 shows the surface hardness of PC/PMMA film obtained at various blend compositions by simultaneous biaxial stretching at various draw ratios. The surface hardness of the neat PC (100/0 PC/PMMA) did not change by stretching. Note that there is no report on the enhancement of the surface hardness by stretching of neat amorphous polymers such as PC, though the surface hardness of the crystallized PET is enhanced by the biaxial stretching due to the increase of the crystallinity [7], and the hardness appears to relate to the yield stress [63] and the yield stress can be increased by stretching. No change of the surface hardness by stretching was observed in the blends of 30/70 and 50/50 PC/PMMA either; the pencil hardness of 30/70 PC/PMMA was 3H at λ = 1.0 and 1.5, and that of 50/50 PC/PMMA was H at λ = 1.0 and 1.5. As shown in Figure 2 and Figure 3, molecular chains are oriented by stretching, but phase inversion did not occur in 30/70 and 50/50 PC/PMMA. The results suggest that the surface hardness cannot be enhanced by only the molecular orientation of PC and PMMA. The most interesting result is that the surface hardness of 70/30 PC/PMMA became harder with an increasing draw ratio; the pencil hardness was 2B at λ = 1.0, HB at λ = 1.5, and 2H at λ = 2.0. By combining the results shown in Figure 10, the surface hardness of the blend at λ = 1.5 and λ = 2.0 was close to that of 50/50 PC/PMMA and 30/70 PC/PMMA, respectively. Note that the surface hardness of PC was enhanced by the surface localization of PMMA due to segregation during the injection-molding; the durometer D hardness increased from 80 to 83 by blending 5% of PMMA [45]. The change of the surface hardness in 70/30 PC/PMMA by biaxial stretching is attributed to the increase of the amount of PMMA at the surface by structure change from the PMMA domain dispersed in the PC matrix to the PC domain dispersed in the PMMA matrix during stretching, due to the phase inversion. This result confirms the phase inversion of the blend by simultaneous biaxial stretching, demonstrated in Figure 5, Figure 6, Figure 7, Figure 8 and Figure 9. Since the critical composition estimated from Figure 10 was 50 wt %, the critical strain for the phase inversion was estimated to be λ = 1.8, in which the pencil hardness was same as that of 50/50 PC/PMMA.

## 4. Conclusions

We found the characteristic structural evolution in the two-phase blends of polycarbonate (PC) and poly(methyl methacrylate) (PMMA) by simultaneous biaxial stretching. The phase structure was enlarged in all directions without the preferred deformation when the symmetric spherical PC domains were dispersed in the PMMA matrix in 30/70 PC/PMMA, while an anisotropic-shaped co-continuous structure was deformed to yield a crosshatched structure in 50/50 PC/PMMA. On the other hand, in 70/30 PC/PMMA the phase inversion occurred from the spherical PMMA domains to the crosshatched PMMA network matrix by in-plane bimodal orientation, due to coalescence and aggregation of the PMMA domains during the biaxial stretching. Owing to the phase inversion, the pencil hardness became harder, from 2B to 2H, due to the increase of the amount of PMMA at the film surface by the simultaneous biaxial stretching. Hence, biaxial stretching of two-phase polymer blends is expected to enhance the surface hardness of packaging films when the phase inversion occurs, for instance.
